# Highly Promiscuous Flavonoid Di-*O*-glycosyltransferases from *Carthamus tinctorius* L.

**DOI:** 10.3390/molecules29030604

**Published:** 2024-01-26

**Authors:** Xiaoyu Xu, Meng Xia, Yang Han, Honghu Tan, Yanying Chen, Xinqi Song, Shijun Yuan, Yifeng Zhang, Ping Su, Luqi Huang

**Affiliations:** 1 Academician Workstation, Research Center for Differentiation and Development of TCM Basic Theory, Jiangxi University of Chinese Medicine, Nanchang 330004, China; 2 State Key Laboratory for Quality Ensurance and Sustainable Use of Dao-di Herbs, National Resource Center for Chinese Materia Medica, Chinese Academy of Chinese Medical Sciences, Beijing 100700, China

**Keywords:** *Carthamus tinctorius* L., flavonoid glycosides, di-*O*-glycosyltransferases

## Abstract

Safflower (*Carthamus tinctorius* L.) has been recognized for its medicinal value, but there have been limited studies on the glycosyltransferases involved in the biosynthesis of flavonoid glycosides from safflower. In this research, we identified two highly efficient flavonoid *O*-glycosyltransferases, *CtOGT1* and *CtOGT2*, from safflower performing local BLAST alignment. By constructing a prokaryotic expression vector, we conducted in vitro enzymatic reactions and discovered that these enzymes were capable of catalyzing two-step *O*-glycosylation using substrates such as kaempferol, quercetin, and eriodictyol. Moreover, they exhibited efficient catalytic activity towards various compounds, including flavones (apigenin, scutellarein), dihydrochalcone (phloretin), isoflavones (genistein, daidzein), flavanones (naringenin, glycyrrhizin), and flavanonols (dihydrokaempferol), leading to the formation of *O*-glycosides. The broad substrate specificity of these enzymes is noteworthy. This study provides valuable insights into the biosynthetic pathways of flavonoid glycosides in safflower. The discovery of *CtOGT1* and *CtOGT2* enhances our understanding of the enzymatic processes involved in synthesizing flavonoid glycosides in safflower, contributing to the overall comprehension of secondary metabolite biosynthesis in this plant species.

## 1. Introduction

*Carthamus tinctorius* L., commonly referred to as safflower, is an herbaceous plant belonging to the Carthamus genus of the Asteraceae family. It has been cultivated for approximately 4500 years and originated from the “New Crescent” region along the eastern Mediterranean coast [1]. Safflower has a rich history in traditional medicine and is known for its therapeutic properties in treating various conditions such as joint injuries and pain. In modern pharmacology, safflower has shown significant effects in improving myocardial ischemia, exerting anti-inflammatory responses, and preventing thrombotic events [2]. Flavonoids are valuable compounds found in safflower, which can be categorized into two major groups. The first group includes special compounds like hydroxysafflor yellow A (HSYA), currently undergoing phase III clinical trials for the treatment of acute ischemic stroke [3]. Safflower yellow injection, containing HSYA as the main component, has obtained approval from the Chinese National Medical Products Administration (NMPA) for treating angina and coronary heart diseases [4]. The second group comprises common compounds such as flavonoids, flavonols, and dihydroflavonoids. Examples of these compounds include quercetin-3-*O*-glucoside (known for its antioxidant, anti-inflammatory, and anticancer properties) [5,6], rutin (possessing anticancer, anti-inflammatory, antibacterial, and antidiabetic effects) [7,8,9], naringenin-7-*O*-glucoside (vasorelaxant, antioxidant, and antidiabetic) [10,11,12], isorhamnetin-3-*O*-glucoside (antiadipogenic, antioxidant, and anti-inflammatory) [13,14,15], and genistin (anticancer, antioxidant, cardioprotective, anti-apoptotic, neuroprotective, hepatoprotective, and antimicrobial) [16]. Xian et al. summarized a total of 24 special category compounds and 43 common compounds that have been identified in safflower (Table 1) [17,18].

Glycosylation is a common post-translational modification in plant biosynthetic pathways that greatly contributes to the structural diversity of plant secondary metabolites [19,20]. Glycosides, categorized as *O*-, *C*-, *S*-, and *N*-glycosides based on the type of glycosidic bond, are formed through this process [21]. In the biosynthesis of glycosylated flavonoids, glycosyltransferases play a vital role. These enzymes utilize nucleotide diphosphate-activated sugar moieties such as UDP-glucose, UDP-xylose, UDP-galactose, UDP-arabinose, UDP-glucuronic acid, UDP-N-acetylglucosamine, and others as sugar donors. They facilitate the formation of region- and stereo-specific glycosidic bonds and belong to the UDP-dependent glycosyltransferase (UGT) family [22,23]. Despite the wide range of flavonoid glycosides present in safflower, only a few UGT genes involved in their biosynthesis have been functionally characterized thus far. The complete elucidation of the biosynthetic pathway for major active compounds like HSYA remains incomplete. Ren et al. demonstrated the glycosylation function of candidate UGTs using safflower corolla protoplasts in vivo for the first time. Overexpression of *CtUGT3* resulted in a significant increase in the content of kaempferol-3-*O*-glucoside, indicating its glycosylation activity towards flavonoids with 3-OH and 7-OH moieties [24]. Qi et al. provided preliminary evidence for the function of *CtUGT4* as a flavonoid-*O*-glycosyltransferase in safflower by demonstrating that its overexpression led to a noteworthy increase in the content of quercetin-3-*O*-rutinoside and a trend of increased quercetin-3-*O*-glycoside content [25]. Although several *O*-glycosyltransferases have been identified in safflower, the aforementioned enzymes did not exhibit significant di-*O*-glycosylation activity.

The application and development of synthetic biology has made significant breakthroughs in achieving the efficient synthesis of active compounds in safflower, which is of great significance for the sustainable utilization of valuable traditional Chinese medicine resources. Here, we reported two highly efficient di-*O*-glycosyltransferases with broad substrate specificity from the safflower, providing references for the elucidation of the biosynthetic pathways of flavonoids within it.

## 2. Results

### 2.1. Screening of Safflower Flavonoid O-Glycosyltransferases

To screen candidate genes encoding *O*-glycosyltransferases in the safflower transcriptome, considering the promiscuity of glycosyltransferase substrates, tBLASTn analysis was performed using the amino acid sequences of *Vitis vinifera*’s glycosyltransferase *VvGT1* and *Arabidopsis thaliana*’s glycosyltransferase *UGT89C1* as reference sequences (E-value < e^−40^) [26,27]. Three candidate genes with complete ORFs were selected from the safflower transcriptome screening and named *CtOGT1* and *CtOGT2*. The ExPASy online server was used to predict the number of amino acids, relative molecular weight, theoretical isoelectric point, molecular formula, and instability coefficient encoded by the candidate genes. The results are shown in Table S1. The three-dimensional protein structure prediction of *CtOGT1* and *CtOGT2* is shown in Figure S1.

### 2.2. Phylogenetic and Multiple Sequence Alignment Analysis of CtOGTs

**Figure 1:**
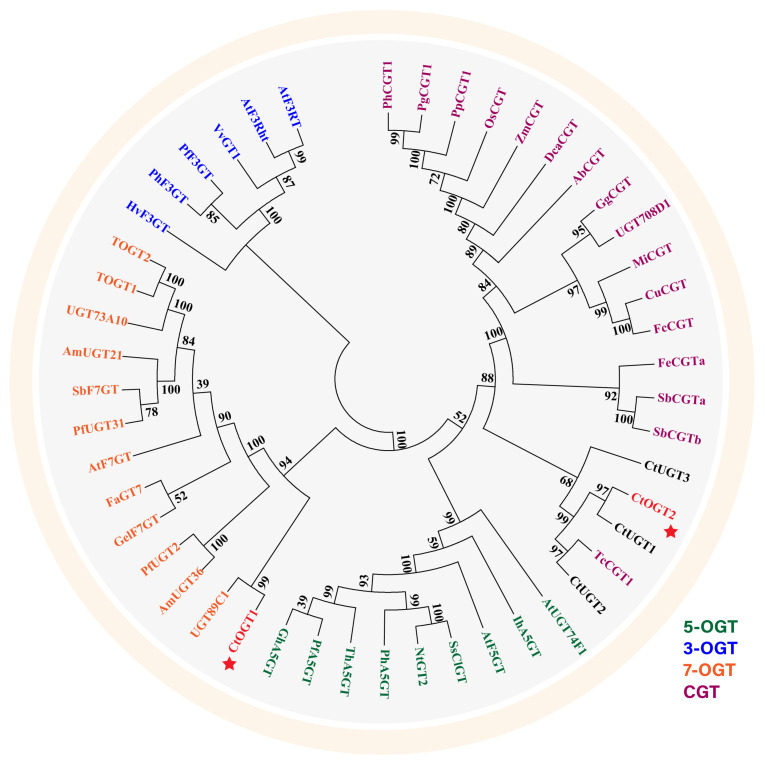
Phylogenetic analyses of *CtOGT1* and *CtOGT2* with 46 other reported glycosyltransferases. PhCGT1 (MK616588, *Phyllostachys heterocycla*); PgCGT1 (MK616592, *Phyllostachys glauca*); PpCGT1 (MK616593, *Phyllostachys prominens*); OsCGT (FM179712, *Oryza sativa*); ZmCGT (NP_001132650, *Zea mays*); DcaCGT (QOD39011, *Dendrobium catenatum*); AbCGT (MN747045, *Aloe barbadensis*); GgCGT (QGL05036, *Glycyrrhiza glabra*); UGT708D1 (LC003312, *Glycine max*); MiCGT (KT200208, *Glycyrrhiza glabra*); CuCGT (LC131334, *Citrus unshiu*); FcCGT (LC131333, *Fortunella crassifolia*); FeCGTa (AB909375, *Fagopyrum esculentum*); SbCGTa (MK894443, *Scutellaria baicalensis*); SbCGTb (MK894444, Scutellaria baicalensis); TcCGT1 (MK644229, *Trollius chinensis*); CtUGT1 (OQ354214, *Carthamus tinctorius* L.); CtUGT2 (OQ354222, *Carthamus tinctorius* L.); CtUGT3 (OQ354223, *Carthamus tinctorius* L.); AtUGT74F1 (NP973682, *Arabidopsis thaliana*); IhA5GT (Q767C8, *Iris hollandica*); AtF5GT (AAM91686, *Arabidopsis thaliana*); SsClGT (AAK54465, *Solanum sogarandinum*); NtGT2 (BAB88935, Nicotiana tabacum); PhA5GT (BAA89009, *Petunia hybrida*); ThA5GT (BAC54093, *Torenia hybrida*); PfA5GT (BAA36421, *Perilla frutescens*); GhA5GT (BAA36423, *Glandularia x hybrida*); UGT89C1 (AAP31923, *Arabidopsis thaliana*); AmUGT36 (BAG16513, *Antirrhinum majus*); PfUGT2 (BAG31951, *Perilla frutescens*); GelF7GT (BAC78438, *Glycyrrhiza echinate*); AtF7GT (AAL90934, *Arabidopsis thaliana*); FaGT7 (Q2V6J9, *Fragaria ananassa*); PfUGT31 (BAG31952, *Perilla frutescens*); SbF7GT (BAA83484, *Scutellaria baicalensis*); UGT73A10 (BAG80536, *Lycium barbarum*); TOGT1 (AAK28303, *Nicotiana tabacum*); TOGT2 (AAK28304, *Nicotiana tabacum*); HvF3GT (CAA33729, *Hordeum vulgare*); PhF3GT (BAA89008, *Petunia hybrida*); PfF3GT (BAA19659, *Perilla frutescens*); VvGT1 (AAB81682, *Vitis vinifera*); AtF3Rht (AAM65321, *Arabidopsis thaliana*); AtF3RT (AAM91139, *Arabidopsis thaliana*). Note: The red stars represent glycosyltransferases characterized from safflower in this article.

**Figure 2:**
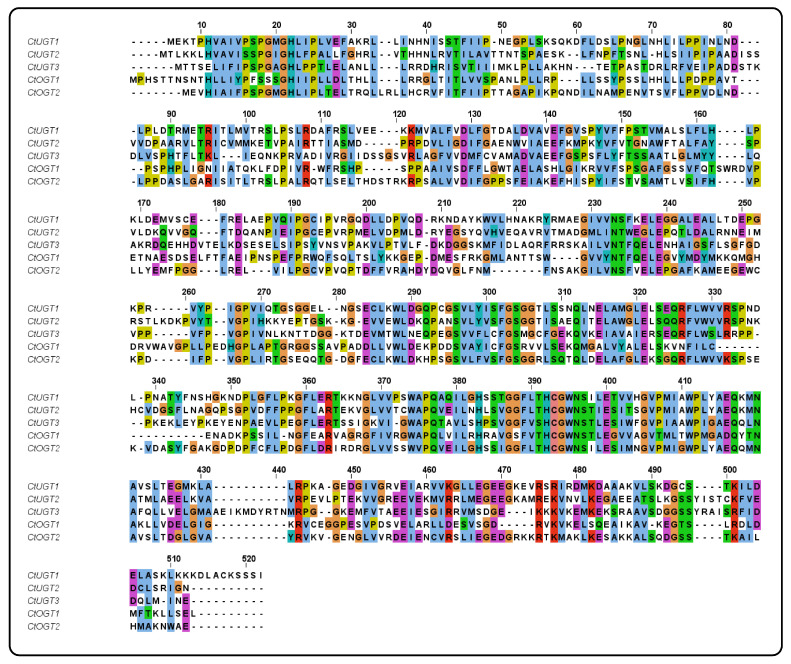
The sequence alignment of genes *CtOGT1*, *CtOGT2*, *CtUGT1*, *CtUGT2*, and *CtUGT3*. Note: Different colors represent the homology of different sequences.

Using MEGA 6.0 software, a systematic evolutionary analysis of 48 glycosyltransferases, including the *CtOGT1* and *CtOGT2* genes, was conducted based on the neighbor-joining method with ClustalW multiple alignments [28]. The phylogenetic analysis revealed that UGTs can be divided into four main branches, including 3-*O*-glycosyltransferases, 5-*O*-glycosyltransferases, 7-*O*-glycosyltransferases, and *C*-glycosyltransferases, as shown in Figure 1. The results indicated that *CtOGT1* clusters with the 7-*O*-glycosyltransferases branch, while *CtOGT2* clusters with *CtUGT1*, *CtUGT2*, and *CtUGT*3, which were already reported to have *O*-glycosyltransferase activity in safflower, as well as *TcCGT1*, which had both *C*- and *O*-glycosyltransferase activity [29]. This predicted result indicates that the genes *CtOGT1* and *CtOGT2* belonged to *O*-glycosyltransferases in safflower. The amino acid sequence identity of *CtOGT1*, *CtOGT2*, *CtUGT1*, *CtUGT2*, and *CtUGT3* was 48.39% (Figure 2).

### 2.3. Cloning and Functional Characterization of CtOGTs

Using safflower cDNA as a template, PCR amplification was performed using high-fidelity polymerase Prime Start Max to obtain the complete ORF fragments of *CtOGT1* and *CtOGT2*, which were 1365 bp and 1383 bp in length, respectively. These fragments were cloned into the pET28-MBP vector, resulting in recombinant expression plasmids pET28-MBP-CtOGT1 and pET28-MBP-CtOGT2. The recombinant plasmids were transformed into BL21(DE3) competent cells, induced with IPTG at 16 °C for 18 h, and purified using His-tag affinity chromatography. The purification of the recombinant pET28-MBP-CtOGT1 and pET28-MBP-CtOGT2 proteins was confirmed using SDS-PAGE analysis, as shown in Figure S2.

To verify the activity of *CtOGT1* and *CtOGT2* towards flavonoid *O*-glycosyltransferases in safflower, we selected flavanols (kaempferol, quercetin), flavones (apigenin, scutellarein), dihydrochalcone (phloretin), isoflavones (genistein, daidzein), flavanones (naringenin, eriodictyol, glycyrrhizin), and flavanonol (dihydrokaempferol) as substrates for detection. The enzymatic reaction system consisted of 50 mM Na_2_HPO_4_-NaH_2_PO_4_ buffer (pH 8.0), 0.2 mM substrate, 0.5 mM UDP-Glc, and 10 μg of purified enzyme. After incubating at 37 °C for 6 h, the reaction was terminated with double the volume of methanol.

**Figure 3:**
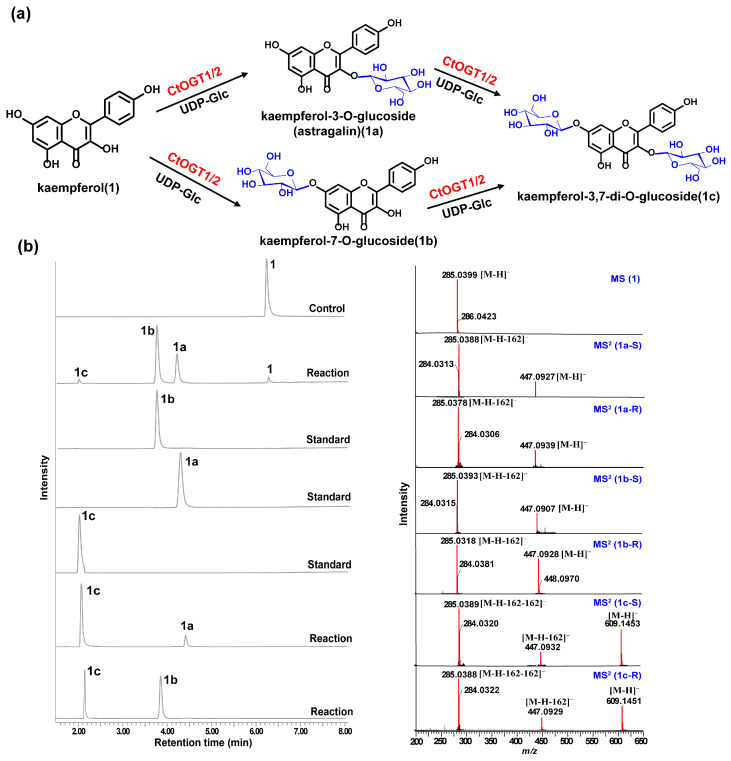
(**a**) LC/MS analysis of *CtOGT1*- and *CtOGT2*-catalyzed product using kaempferol as the substrate. Compounds **1**, **1a**, **1b**, and **1c** represent kaempferol, kaempferol-7-*O*-glucoside, kaempferol-3-*O*-glucoside, and kaempferol-3,7-di-*O*-glucoside, respectively. (**b**) (−)-ESI-MS spectra of 1, (−)-ESI-MS2 spectra of **1a**, **1b**, and **1c**. S: standard, R: reaction.

**Figure 4:**
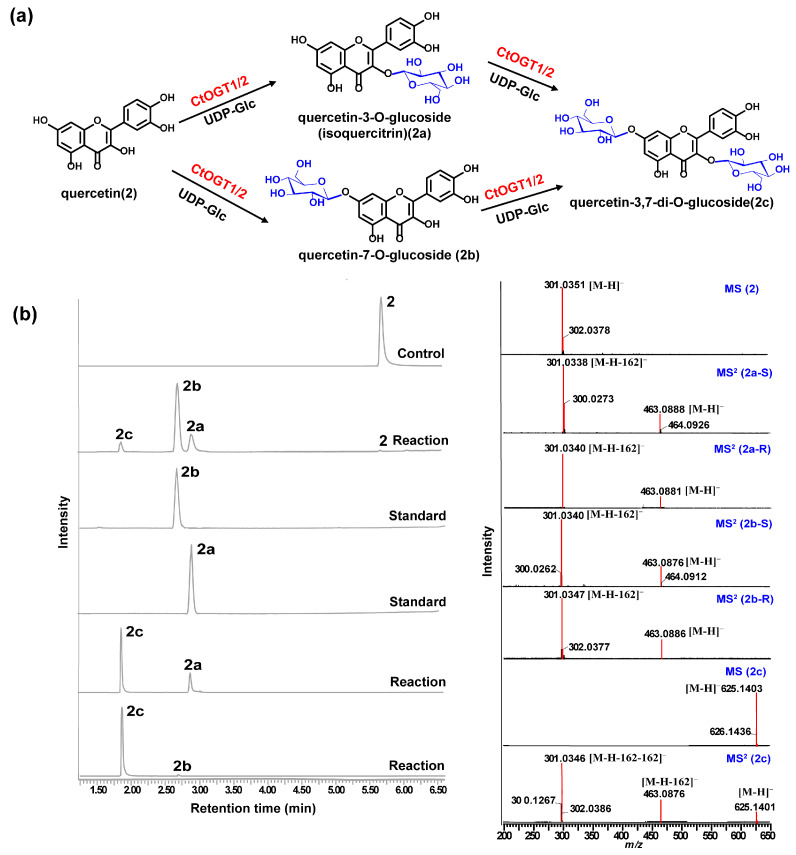
(**a**) LC/MS analysis of *CtOGT1-* and *CtOGT2*-catalyzed product using quercetin as the substrate. Compounds **2**, **2a**, **2b**, and **2c** represent quercetin, isoquercitrin, quercetin-7-*O*-glucoside, and quercetin-3,7-di-*O*-glucoside respectively. (**b**) (−)-ESI-MS spectra of **2** and **2c**, (−)-ESI-MS^2^ spectra of **2a**, **2b**, and **2c**. S: standard, R: reaction.

**Figure 5:**
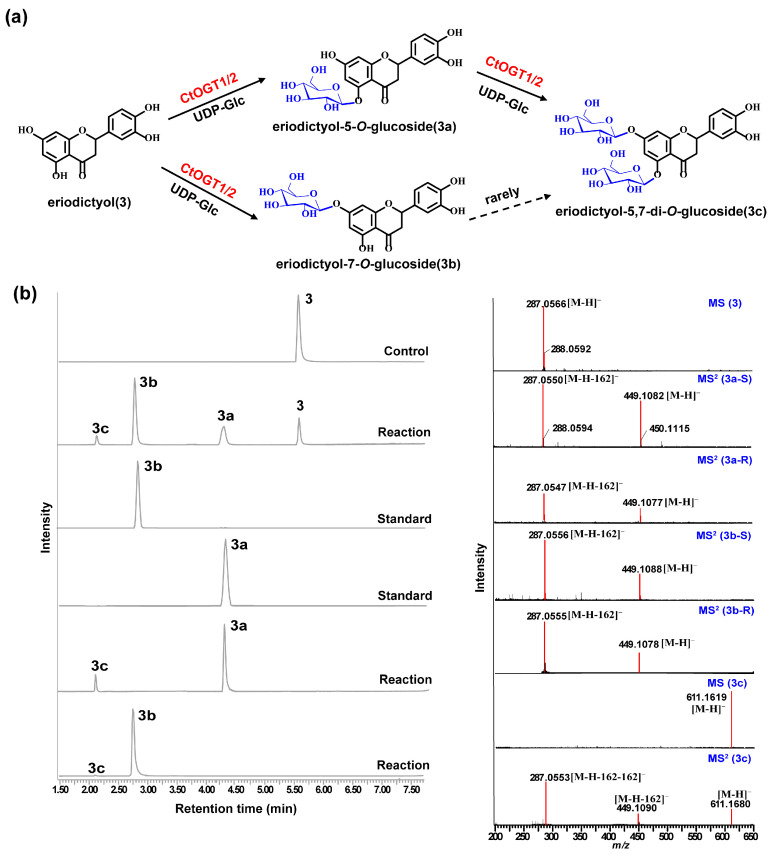
(**a**) LC/MS analysis of *CtOGT1-* and *CtOGT2*-catalyzed product using eriodictyol as the substrate. Compounds **3**, **3a**, **3b**, and **3c** represent eriodictyol, eriodictyol-5-*O*-glucoside, eriodictyol-7-*O*-glucoside, and eriodictyol-5,7-di-*O*-glucoside, respectively. (**b**) (−)-ESI-MS spectra of **3** and **3c**, (−)-ESI-MS^2^ spectra of **3a**, **3b**, and **3c**. S: standard, R: reaction.

Through liquid chromatography combined with mass spectrometry (LC/MS) analysis and comparison with the standard references, the results showed that, compared to the control group, the genes *CtOGT1* and *CtOGT2* could respectively catalyze the conversion of kaempferol 3-OH and 7-OH into kaempferol-7-*O*-glucoside and kaempferol-3-*O*-glucoside (astragalin) (**1a** and **1b**). Additionally, the presence of kaempferol-3,7-di-*O*-glucoside (**1c**) was detected. *CtOGT1* exhibited better catalytic activity for di-*O*-glycosylation compared to *CtOGT2*. When quercetin was used as a substrate, both *CtOGT1* and *CtOGT2* catalyzed the conversion of quercetin 3-OH and 7-OH into isoquercitrin, quercetin-7-*O*-glucoside, and quercetin-3,7-di-*O*-glucoside (**2a**, **2b** and **2c**). When eriodictyol was used as a substrate, *CtOGT1* and *CtOGT2* catalyzed the formation of eriodictyol-5-*O*-glucoside, eriodictyol-7-*O*-glucoside, and eriodictyol-5,7-di-*O*-glucoside (**3a**, **3b** and **3c**), as shown in Figure 3, Figure 4 and Figure 5. Then, enzymatic reactions were conducted in vitro using substrates **1a**, **1b**, **2a**, **2b**, **3a**, and **3b**. As a result, it was found that compound **1c** could be detected when using **1a** and **1b** as substrates, indicating that *CtOGT1* and *CtOGT2* can catalyze the formation of **1c** from **1**, **1a**, and **1b**, respectively. Similarly, compound **2c** could also be detected when using **2**, **2a**, and **2b** as substrates. However, when using **3b** as a substrate, only a small amount of **3c** was detected. This indicated that *CtOGT1* and *CtOGT2* could catalyze the formation of **3c** from **3** and **3a**, but it was difficult to catalyze the formation of **3c** from **3b**. Furthermore, *CtOGT1* and *CtOGT2* exhibited better *O*-glycosyltransferase activity towards substrates apigenin, scutellarein, phloretin, genistein, daidzein, naringenin, glycyrrhizin, and dihydrokaempferol (**4**–**11**), indicating a broad substrate specificity of *CtOGT1* and *CtOGT2*, as shown in Figures S3–S10 (all reactions were plotted using *CtOGT1* as an example). These in vitro enzymatic activity results were consistent with the predicted result, indicating that the genes *CtOGT1* and *CtOGT2* belonged to *O*-glycosyltransferases in safflower.

## 3. Discussion

Safflower contains quinone chalcone compounds with unique structures and significant therapeutic activities in cardiovascular and cerebrovascular diseases. Examples of these compounds include HSYA, safflor yellow A, and carthamin. These compounds are exclusively found in safflower and belong to the *C*-glycoside class. Therefore, the biosynthesis pathway of these special flavonoids in safflower has attracted researchers’ attention [17]. Common flavonoid compounds in safflower are also found in many other species and display various activities. These include kaempferol, naringenin, quercetin, apigenin, and luteolin, among others [30].

Xie et al. discovered a novel glycosyltransferase, *UGT73AE1*, from safflower. *UGT73AE1* demonstrated the ability to glucosylate 19 structurally diverse acceptor molecules and generate *O*-, *S*-, and *N*-glycosides, making it the first reported plant glycosyltransferase with tri-functional activity [31]. Qi et al. found that *CtUGT4* can catalyze the formation of quercetin-7-*O*-glucoside from quercetin and naringenin-3-*O*-glucoside from naringenin in vitro. Additionally, a range of new products were generated using various compounds as substrates, including flavones, flavonols, flavanones, chalcones, and chalcone glycosides [25]. Studies have shown that *CtUGT3* catalyzes the glucosylation of flavones (apigenin), flavanones (naringenin), flavonols (kaempferol, quercetin, and isorhamnetin), and chalcones (naringenin chalcone), forming their respective *O*-glucosides. Furthermore, when flavonols were used as aglycones in vitro, *CtUGT3* exhibited both 7-OH and 3-OH glycosylation activities simultaneously [24]. The most abundant flavonoid glycosides in plants are flavone *O*- or *C*-glycosides and flavonol *O*-glycosides, with glycosides at the 3-OH or 7-OH positions being the most common. According to reports, the genes *UGT78K1* and *Fh3GT1* both possess the activity of flavonoid 3-*O*-glycosyltransferase [32,33]. Wang et al. discovered a highly selective and donor-diverse 3-*O*-glycosyltransferase, *Sb3GT1* (*UGT78B4*), from *Scutellaria* baicalensis. *Sb3GT1* was capable of accepting five sugar donors (UDP-Glc/-Gal/-GlcNAc/-Xyl/-Ara) to catalyze the 3-*O*-glycosylation reaction of 17 flavonoid compounds, with conversion rates exceeding 98% [34]. *CsUGT75L12* encoded a flavonoid 7-*O*-glucosyltransferase, which can specifically transfer the glucose moiety from UDP-glucose to the 7-hydroxyl position of flavonoids, generating the corresponding 7-*O*-glucosides. Furthermore, the expression pattern of the *CsUGT75L12* gene was consistent with the accumulation pattern of 7-*O*-glucosides and 7-*O*-neohesperidoside in tea plants, indicating its involvement in the biosynthesis of bitter flavonoid 7-*O*-neohesperidoside [35]. In addition, genes such as *NpUGT6*, *UGT73CD1*, *TwUGT3*, and *UGT71E5* have been found to exhibit catalytic activity in the *O*-glycosylation of various flavonoid compounds [36,37,38,39]. However, there is currently a relatively limited amount of research on glycosyltransferases in safflower. Despite the presence of abundant flavonoid compounds in safflower that are typically conjugated with sugar moieties to form glycosides, our understanding of the specific glycosyltransferases involved in catalyzing these reactions remains limited. Therefore, further in-depth studies on glycosyltransferases in safflower are necessary.

In this study, *CtOGT1* and *CtOGT2*, as newly discovered *O*-glycosyltransferases in safflower, have shown significant versatility and catalytic activity. They exhibit *O*-glycosylation activity towards a wide range of flavonoid compounds, including flavanols (kaempferol, quercetin), flavones (apigenin, scutellarein), dihydrochalcone (phloretin), isoflavones (genistein, daidzein), flavanones (naringenin, eriodictyol, glycyrrhizin), and flavanonol (dihydrokaempferol). Notably, *CtOGT1* and *CtOGT2* demonstrate an ability to catalyze the addition of glucose moieties to specific hydroxyl positions (such as 7-OH, 3-OH, and 5-OH) of various flavonoid structures. By catalyzing the formation of specific glycosides, such as kaempferol-7-*O*-glucoside, astragalin, kaempferol-3,7-di-*O*-glucoside, quercetin-7-*O*-glucoside, isoquercitrin, quercetin-3,7-di-*O*-glucoside, eriodictyol-7-*O*-glucoside, eriodictyol-5-*O*-glucoside, and eriodictyol-5,7-di-*O*-glucoside, *CtOGT1* and *CtOGT2* contribute to the synthesis of diverse flavonoid glycosides with medicinal value. These glycosides play crucial roles in various biological processes and contribute to the unique properties, taste, and health benefits associated with safflower. The discovery and characterization of *CtOGT1* and *CtOGT2* shed light on the glycosylation pathways involved in safflower’s flavonoid metabolism. These findings not only enhance our understanding of secondary metabolite biosynthesis in safflower but also provide valuable insights into the regulation of bitterness and other sensory attributes in safflower-based products.

Synthetic biology has played a crucial role in the research of flavonoid *O*-glucosides. Through genetic engineering techniques, glucosyltransferases (GTs) from various sources can be introduced into host microorganisms to enable the enzymatic glucosylation of flavonoid compounds. Common expression hosts for these GTs include *E. coli* and *Saccharomyces cerevisiae*. By adjusting reaction conditions and substrate concentrations, the efficient and controllable synthesis of flavonoid *O*-glucosides can be achieved. Flavonoid 7-*O*-glucosides exhibit various biological activities; however, some are not abundant in nature. Therefore, a method for producing flavonoid 7-*O*-glucosides was investigated. *E. coli*-expressing tobacco-derived glucosyltransferase (*Ec-NtGT2*) converted several flavonoid compounds (apigenin, luteolin, quercetin, kaempferol, and naringenin) into their respective 7-*O*-glucosides, with conversion rates ranging from 67% to 98%. In scaled-up production, *Ec-NtGT2* yielded 24 mg/L of apigenin 7-*O*-glucoside, 41 mg/L of luteolin 7-*O*-glucoside, 118 mg/L of quercetin 7-*O*-glucoside, 40 mg/L of kaempferol 7-*O*-glucoside, and 75 mg/L of naringenin 7-*O*-glucoside through the sequential addition of substrates within 4–9 h. The conversion rates for apigenin, luteolin, quercetin, kaempferol, and naringenin were 97%, 72%, 77%, 98%, and 96%, respectively. These results indicated that *Ec-NtGT2* was a simple and efficient biotransformation system for the production of flavonoid 7-*O*-glucosides [40]. Zhao et al. achieved de novo biosynthesis of the isoflavones genistein in *E. coli*. The results showed that the LCA2G-LNR30R-LGN43 three-strain system was more suitable for synthesizing it. Under the optimal inoculation ratio of 2:1:4, the production of genistein reached 35.1 mg/L [41]. Li et al. biosynthesized baicalin and scutellarein in *E. coli*, with yields of 23.6 mg/L and 106.5 mg/L, respectively [42]. Liu et al. established a yeast cell factory for the production of breviscapine, with a titer reaching 108 mg/L [43]. Nielsen et al. constructed a de novo synthesis yeast cell factory for isoflavones biosynthesis, producing the core chemical scaffold genistein, with titers of 72.8 mg/L for puerarin and 73.2 mg/L for daidzein, by introducing glycosyltransferases in the engineered strains [44]. Synthetic biology provides a sustainable and efficient approach to the synthesis of flavonoid *O*-glucosides. It can be utilized not only for large-scale production but also for the targeted synthesis of specific structures of flavonoid *O*-glucosides. This is of great significance in studying the biological activities of flavonoid compounds and drug development, providing new insights and tools for further research and applications in related fields. As a precious and highly valued traditional Chinese medicine with immense medicinal value, it is of the utmost importance to decipher the biosynthetic pathways of the major compounds in safflower and achieve efficient heterologous production.

In summary, we successfully characterized two new di-*O*-glycosyltransferases, *CtOGT1* and *CtOGT*2, from *Carthamus tinctorius* L., a medicinal plant. It demonstrated efficient catalytic activity in di-*O*-glycosylation of at least seven substrates containing flavonols (kaempferol, quercetin), flavone (scutellarein), dihydrochalcone (phloretin), and flavanones (naringenin, eriodictyol, glycyrrhizin), as well as mono-*O*-glycosylation of at least 11 flavonoid compounds. This study provides valuable insights for future research on the biosynthetic pathways of flavonoids in safflower.

## 4. Materials and Methods

### 4.1. Plant Materials

Safflower (*Carthamus tinctorius* L.) samples were obtained from the Henan Academy of Agricultural Sciences. The collected samples were washed with sterile deionized water and subsequently frozen in liquid nitrogen. They were then stored at −80 °C until RNA extraction.

### 4.2. Phylogenetic Analysis and Sequence Alignment

To perform sequence comparisons and analysis, MEGA 6.0 software was utilized. Subsequently, a phylogenetic tree was constructed using the neighbor-joining method for cluster analysis, incorporating *CtOGT1* and *CtOGT2* along with other glycosyltransferase sequences. The amino acid sequences of *CtOGT1*, *CtOGT2*, *CtUGT1*, *CtUGT2*, and *CtUGT3* were aligned using DNAMAN 8.0 software for multiple sequence alignment [45].

### 4.3. Molecular Cloning

To identify potential OGT genes, a BLAST search was performed on the transcriptome database of *Carthamus tinctorius* L., using *Vitis vinifera*’s glycosyltransferase *VvGT1* and *Arabidopsis thaliana*’s glycosyltransferase *UGT89C1* as reference sequences. This search led to the selection of two candidate genes, namely *CtOGT1* and *CtOGT2*, which possessed complete open reading frames (ORFs). For detailed sequence information, please refer to Table S2. The ExPASy online server (http://web.expasy.org/protparam/, accessed on 8 May 2023) was utilized to predict various characteristics encoded by *CtOGT1* and *CtOGT*2. Furthermore, the protein structures of *CtOGT1* and *CtOGT2* were predicted using AlphaFold (https://alphafold.com/, accessed on 10 May 2023).

Total RNA was isolated from safflower samples using the TranZolTM kit (Transgen Biotech, Beijing, China). Reverse transcription was performed to convert the extracted RNA into cDNA using the TransScript II One-Step gDNA Removal and cDNA Synthesis SuperMix (Transgen Biotech, China). Specific primers were designed for amplifying the candidate *O*-glycosyltransferase genes. The amplification reactions were carried out using 2× Prime Start Max Enzyme under the following conditions: 5 min at 98 °C; 15 s at 98 °C, 15 s at 55 °C, 50 s at 72 °C for 40 cycles; and 7 min at 72 °C. The resulting PCR products were then ligated into the pEASY-Blunt Zero Cloning Kit vector (TsingKe, Beijing, China) and sequenced to confirm their integrity. Subsequently, using a positive plasmid as a template, the target gene with *BamH*I restriction sites was cloned. The *BamH*I-digested pET28a-MBP vector and the target gene fragment were seamlessly joined together using seamless cloning technique. The constructed recombinant plasmids were transformed into *E. coli* BL21 (DE3) cells (Transgen Biotech) for heterologous expression on LB plates supplemented with kanamycin (50 mg/L). Detailed primer sequences used in this study can be found in Table 2.

### 4.4. Expression and Purification of CtOGT1 and CtOGT2

The recombinant expression plasmids were separately transformed into BL21(DE3) competent cells. After the appearance of bacterial colonies, single clones were picked and expanded for cultivation until the OD_600_ reached 0.6–0.7. Then, isopropyl-β-d-thiogalactopyranoside (IPTG) was added, and the cells were induced at a low temperature of 16 °C for 18 h. Following induction, the cells were harvested through centrifugation at 8000 rpm for 5 min at 4 °C. The resulting cell pellet was resuspended in 5 mL of lysis buffer (50 mM Na_2_HPO_4_-NaH_2_PO_4_, pH 8.0, 300 mM NaCl) and sonicated in an ice bath. Subsequent removal of cell debris was accomplished by centrifugation at 12,000 rpm for 10 min at 4 °C, with the supernatant collected for further use. The collected supernatant was mixed with Ni-NTA resin (DP101, Transgen Biotech, China) and incubated with agitation for 1.5 h at 4 °C. Using a 50 mM Na_2_HPO_4_-NaH_2_PO_4_ buffer (pH 8.0) containing 20 mM imidazole and 300 mM NaCl, the resin was washed with 10–20 mL of buffer. Elution was performed sequentially using a range of imidazole concentrations in the buffer salt (20/50/150/200/250 mM), with 2–5 mL fractions collected at each step. Next, 15 μL of collected fractions was mixed with 5 μL of 4× protein loading buffer (LABLEAD, Beijing, China) for SDS-PAGE gel electrophoresis. The fractions containing the target protein were concentrated using a 30 kDa ultrafiltration tube (15 mL) at 4000 rpm to a final volume of 500 μL. The concentrated protein was exchanged with a 50 mM Na_2_HPO_4_-NaH_2_PO_4_ buffer salt (pH 8.0) to replace the imidazole elution solution. The purified *CtOGT1* and *CtOGT2* enzymes were stored at −80 °C in the presence of glycerol for long-term storage. The protein concentration was determined using the Protein Quantitative Kit (TransGen Biotech), employing bovine serum albumin (BSA) as the standard.

### 4.5. Enzyme Activity Assay

To assess the functionality of *CtOGT1* and *CtOGT2*, various substrates (kaempferol, quercetin, apigenin, scutellarein, phloretin, genistein, daidzein, naringenin, eriodictyol, glycyrrhizin, and dihydrokaempferol) were employed. UDP-Glc served as the sugar donor in the glycosylation reactions. The reaction mixture comprised 0.5 mM UDP-Glc, 0.1 mM substrate, protein, and a buffer containing 50 mM Na_2_HPO_4_-NaH_2_PO_4_ and 300 mM NaCl (pH 8.0), making a final volume of 500 μL. Incubation of the reactions took place at 37 °C for 6 h, followed by termination with double the volume of methanol. Supernatants collected after centrifugation at 15,000 rpm for 15 min were subjected to LC/MS analysis. Each substrate underwent three parallel reactions.

### 4.6. General Procedures

Compounds **1**–**11**, **1a**, **1b**, **2a**, **2b**, **3a**, and **3b** were acquired from Beite Renkang (Beijing, China), while UDP-glucose was obtained from Sigma-Aldrich (St. Louis, MO, USA). An UPLC-QTOF-MS system (Waters Technologies, Milford, MA, USA) was employed to analyze all products. The Acquity UPLC utilized a T3 column (2.1 mm × 100 mm, 1.8 μm particle size; Waters Technologies) held at 40 °C. The mobile phase consisted of water containing 0.1% (*v*/*v*) formic acid (A) and acetonitrile containing 0.1% (*v*/*v*) formic acid (B). A linear gradient elution program was applied with the following conditions: 0 min, 90% A; 1 min, 80% A; 3 min, 80% A; 4 min, 75% A; 8 min, 30% A; 9 min, 5% A; 12 min, 5% A; 12.2 min, 90% A; 15.2 min, 90% A. For MS analysis, the effluent was introduced into the ESI source of the mass spectrometer at a flow rate of 0.4 mL/min using a T-union splitter. The mass spectrometer operated in (−)-ESI mode.

## Figures and Tables

**Table 1:** The flavonoid compounds in safflower.

No.	Compounds	Molecular Formula	Type
1	Acacetin	C_16_H_12_O_5_	Flavonoid
2	Acacetin-7-*O*-*β*-d-glucuronide (Tilianin)	C_22_H_22_O_10_	Flavonoid
3	Acacetin-7-*O*-alpha-l- rhamnopyranoside	C_22_H_22_O_9_	Flavonoid
4	Acacetin-7-*O*-*β*-d-apiofuranosyl (1→6)-*O*-*β*-d-glucoside	C_27_H_30_O_14_	Flavonoid
5	Apigenin	C_15_H_10_O_5_	Flavonoid
6	Apigenin-6,8-di-C-*β*-d-glucopyranoside	C_27_H_30_O_15_	Flavonoid
7	6-Hydroxyapigenin (Scutellarein)	C_15_H_10_O_6_	Flavonoid
8	Luteolin	C_15_H_10_O_6_	Flavonoid
9	Cynaroside	C_21_H_20_O_11_	Flavonoid
10	Luteolin-7-*O*-(6″-*O*-acetyl)-*β*- glucopyranoside	C_23_H_22_O_12_	Flavonoid
11	Naringenin	C_15_H_12_O_5_	Flavonoid
12	Naringin	C_27_H_32_O_14_	Flavonoid
13	Scutellarin	C_21_H_18_O_12_	Flavonoid
14	Kaempferol	C_15_H_10_O_6_	Flavonol
15	Kaempferide	C_16_H_12_O_6_	Flavonol
16	Kaempferol-3-*O*-*β*-d-glucoside (Astragalin)	C_21_H_20_O_11_	Flavonol
17	Kaempferol-3-*O*-*β*-d-rutinoside	C_27_H_30_O_15_	Flavonol
18	kaempferol-3-*O*-*β*-d-glucopyranosyl- 7-*O*-*β*-d-glucopyranoside	C_27_H_30_O_16_	Flavonol
19	Kaempferol-3-*O*-*β*-sophoroside (Sophoraflavonoloside)	C_27_H_30_O_16_	Flavonol
20	6-Hydroxykaempferol	C_15_H_10_O_7_	Flavonol
21	6-Hydroxykaempferol-3-*O*-*β*-glucoside	C_21_H_20_O_12_	Flavonol
22	6-Hydroxykaempferol-7-*O*-*β*-glucoside	C_21_H_20_O_12_	Flavonol
23	6-Hydroxykaempferol-3,6-di-O-*β*- glucoside	C_27_H_30_O_17_	Flavonol
24	6-Hydroxykaempferol-3,7-di-*O*-*β*- glucoside	C_27_H_30_O_17_	Flavonol
25	6-Hydroxykaempferol-6,7-di-*O*-*β*- glucoside	C_27_H_30_O_17_	Flavonol
26	6-Hydroxykaempferol-3,6,7-tri-*O*-*β*- glucoside	C_33_H_40_O_22_	Flavonol
27	6-Hydroxykaempferol-3,6-di-*O*-*β*-glucoside-7-*O*-*β*-glucuronide	C_33_H_38_O_23_	Flavonol
28	6-Hydroxykaempferol-3-*O*-*β*- rutinoside-6-*O*-*β*-7-glucoside	C_33_H_40_O_21_	Flavonol
29	6-Hydroxykaempferol-3-*O*-*β*-rutinoside	C_27_H_30_O_16_	Flavonol
30	Quercetin	C_15_H_14_O_9_	Flavonol
31	Quercetin-3-*O*-*β*-d-glucoside (Isoquercetin)	C_21_H_20_O_12_	Flavonol
32	Quercetin-3-*O*-*β*-d-galactosid (Hyperoside)	C_21_H_20_O_12_	Flavonol
33	Quercetin-7-*O*-*β*-glucoside	C_21_H_20_O_12_	Flavonol
34	Quercetin-3,7-di-*O*-*β*-glucoside	C_27_H_30_O_17_	Flavonol
35	Quercetin-3-*O*-*α*-l-rhamnoside-7-*O*-*β*- glucuronide	C_27_H_30_O_16_	Flavonol
36	Rutin	C_27_H_30_O_16_	Flavonol
37	Myricetin	C_15_H_10_O_8_	Flavonol
38	Eriodictyol	C_15_H_12_O_6_	Flavanone
39	(2*S*)-4′,5,6,7-tetrahydroxy flavanone 6-*O*-*β*-d-glucoside	C_21_H_27_O_10_	Favanone
40	(2*R*)-5,6,7,4′-tetrahydroxyflavanone- 6,7-diglucoside	C_27_H_32_O_16_	Flavanone
41	(2*S*)-5,6,7,4′-tetrahydroxyflavanone- 6,7-diglucoside	C_27_H_32_O_16_	Flavanone
42	Saffloflavonesides A	C_21_H_18_O_9_	Flavanone
43	Saffloflavonesides B	C_21_H_18_O_9_	Flavanone
44	Hydroxysafflor yellow A (Safflomin A)	C_27_H_32_O_16_	Quinochalcones
45	Hydroxysafflor yellow B (Safflomin B)	C_27_H_32_O_16_	Quinochalcones
46	Hydroxysafflor yellow A-4′-*O*-*β*-d- glucopyranosid	C_33_H_42_O_21_	Quinochalcones
47	3′-hydroxyhydroxysafflor yellow A	C_27_H_32_O_17_	Quinochalcones
48	Safflomin C	C_30_H_30_O_14_	Quinochalcones
49	Isosafflomin C	C_27_H_29_O_15_	Quinochalcones
50	Methylsafflomin C	C_28_H_31_O_15_	Quinochalcones
51	Methylisosafflomin C	C_28_H_31_O_15_	Quinochalcones
52	Anhydrosafflor yellow B	C_48_H_52_O_26_	Quinochalcones
53	Safflor yellow A	C_27_H_30_O_15_	Quinochalcones
54	Safflor yellow B	C_48_H_54_O_27_	Quinochalcones
55	Cartormin	C_27_H_29_O_13_N	Quinochalcones
56	Isocartormin	C_27_H_29_O_13_N	Quinochalcones
57	Tinctormine	C_27_H_31_O_14_N	Quinochalcones
58	Saffloquinoside A	C_27_H_29_O_15_	Quinochalcones
59	Saffloquinoside B	C_34_H_38_O_17_	Quinochalcones
60	Saffloquinoside C	C_27_H_30_O_15_	Quinochalcones
61	Saffloquinoside D	C_27_H_31_O_16_	Quinochalcones
62	Saffloquinoside E	C_30_H_29_O_14_	Quinochalcones
63	Carthamine	C_43_H_42_O_22_	Quinochalcones
64	Hydroxyethylcarthamin	C_45_H_46_O_23_	Quinochalcones
65	Precarthamin	C_44_H_43_O_23_	Quinochalcones
66	Neocarthamin	C_21_H_22_O_11_	Quinochalcones
67	Carthamone	C_21_H_20_O_11_	Quinochalcones

**Table 2:** The design of primer sequences for candidate genes.

Gene	Primer	Sequence (5′-3′)
CtOGT1	CtOGT1-F	ATGCCTCACTCAACCACCAAC
	CtOGT1-R	TTAGAGCTCCGATAGAAGCTT
	CtOGT1-*BamH*I-F	TTAGAGCTCCGATAGAAGCTT ATGCCTCACTCAACCACCAAC
	CtOGT1-*BamH*I-R	ACGGAGCTCGAATTCGGATCC TTAGAGCTCCGATAGAAGCTT
CtOGT2	CtOGT2-F	ATGGAAGTTCACATAGCCATT
	CtOGT2-R	TTACTCAGCCCACTTTTTAGC
	CtOGT2-*BamH*I-F	ATGGAAGTTCACATAGCCATT TTAGAGCTCCGATAGAAGCTT
	CtOGT2-*BamH*I-R	ACGGAGCTCGAATTCGGATCC TTACTCAGCCCACTTTTTAGC

## Data Availability

Data are contained within the article or Supplementary Materials.

## Supplementary Materials

The following supporting information can be downloaded at: https://www.mdpi.com/article/10.3390/molecules29030604/s1, Table S1: Physicochemical analysis of the amino acids encoded by *CtOGT1* and *CtOGT2*; Table S2: The sequences of *CtOGT1* and *CtOGT2* in *Carthamus tinctorius* L.; Figure S1: (a) The three-dimensional protein structure prediction of *CtOGT1*; (b) the three-dimensional protein structure prediction of *CtOGT2*; Figure S2: SDS-PAGE analysis of recombinant *CtOGT1* and *CtOGT2* purified via affinity chromatography. Note: M: Marker; Figure S3: LC-MS analysis of *CtOGT1*- and *CtOGT2*-catalyzed product using apigenin as the substrate; Figure S4: LC-MS analysis of *CtOGT1-* and *CtOGT2*-catalyzed product using scutellarein as the substrate; Figure S5: LC-MS analysis of *CtOGT1-* and *CtOGT2*-catalyzed product using phloretin as the substrate; Figure S6: LC-MS analysis of *CtOGT1-* and *CtOGT2*-catalyzed product using genistein as the substrate; Figure S7: LC-MS analysis of *CtOGT1-* and *CtOGT2*-catalyzed product using daidzein as the substrate; Figure S8: LC-MS analysis of *CtOGT1-* and *CtOGT2*-catalyzed product using naringenin as the substrate; Figure S9: LC-MS analysis of *CtOGT1*- and *CtOGT2*-catalyzed product using glycyrrhizin as the substrate; Figure S10: LC-MS analysis of *CtOGT1-* and *CtOGT2*-catalyzed product using dihydrokaempferol as the substrate.
