# Supplementary material for: Highly Promiscuous Flavonoid Di-O-glycosyltransferases from Carthamus tinctorius L
Source: Molecules. 2024 Jan 26;29(3):604. doi: 10.3390/molecules29030604 (PMC10856022; doi:10.3390/molecules29030604)
Supplement: Supplementary file 1 [file molecules-29-00604-s001.zip › molecules-2831889-supplementary.pdf]

Table S1. Physicochemical analysis of the amino acids encoded by *CtOGT1* and *CtOGT2*.

	CtOGT1	***CtOGT2***
Number of amino acids	454	460
Molecular weight	49.87	50.64
Theoretical pI	5.73	5.82
Instability index	41.65	43.53
Formula	$C_{2257}H_{3518}N_{594}O_{653}S_{14}$	$C_{2292}H_{3589}N_{601}O_{654}S_{19}$
Aliphatic index	94.45	93.85

Table S2. The sequences of *CtOGT1 and CtOGT2* in *Carthamus tinctorius L.*.

**Gene**	**Nucleotide sequence**
CtOGT1	ATGCCTCACTCAACCACCAACAGCAACACACATTTACTAATCTACCCTTT
	TTCTAGCTCCGGCCACATCATCCCTTTACTCGACCTCACCCATCTCCTCCT
	CCGCCGTGGCCTCACCATCACCCTCGTCGTCTCTCCGGCCAACCTCCCTCT
	CCTCCGCCCTCTCCTCTCTTCCTACCCCTCTTCCCTCCACCACCTTCTCCTC
	CCCGATCCCCCCGCCGTCACCCCCTCCCCCCATCCCCTTATCGGCAATAT
	CATCGCCACCCAAAAACTCTTCGATCCCATCGTTCGATGGTTTCGGTCCC
	ACCCGTCACCGCCGGCCGCCATCGTCTCCGACTTCTTTCTCGGGTGGACC
	GCGGAACTCGCTTCCCACCTCGGGATCAAACGGGTGGTGTTTTCGCCTTC
	CGGTGCTTTCGGTTCTTCCGTTTTCCAAACGTCATGGCGAGACGTGCCGG
	AGACTAACGCCGAAAGCGACAGCGAATTGTTTACGTTCGCAGAAATCCC
	AAACTCGCCGGAATTTCCGCGGTGGCAGTTCTCGCAGCTGACGAGTCTTT
	ATAAGAAAGGCGAACCGGATATGGAATCTTTTCGGAAGGGAATGTTGGC
	GAACACGACGAGCTGGGGAGTCGTGTACAACACATTCCAAGAGTTGGAA
	GGGGTTTATATGGATTACATGAAGAAACAAATGGGCCATGATCGGGTTT
	GGGCCGTGGGCCCGTTACTTCCTGAGGATCATGGCCCACTAGCTCCAACC
	GGACGAGGTGGGTCAAGTGCAGTGCCCGCTGATGACCTCCTCGTCTGGTT
	GGACGAAAAGCCTGACGACTCGGTTGCTTATATATGTTTTGGCAGCCGAG
	TCGTCTTAAGCGAGAAGCAAATGGGTGCACTGGTGTATGCACTCGAGCTT
	AGTAAAGTTAATTTTATCTTGTGTGAGAATGCAGACAAGCCGAGCTCGAT
	CCTTAATGGGTTCGAGGCTCGGGTTGCTGGTCGAGGGTTCATCGTCAGAG
	GATGGGCCCCGCAATTGGTTATACTGAGACATCGAGCCGTCGGCTCGTTT
	GTGACTCACTGTGGGTGGAACTCGACGTTGGAAGGAGTTGTGGCGGGTG
	TGACTATGTTGACGTGGCCAATGGGTGCGGACCAATACACAAATGCTAA
	GTTATTGGTTGACGAATTGGGTATCGGGAAACGAGTTTGCGAGGGTGGG
	CCAGAGAGCGTCCCTGACTCGGTCGAGTTGGCTCGGTTGTTGGATGAGTC
	AGTGAGTGGTGATAGAGTTAAAGTAAAGGAGCTAAGCCAAGAAGCAAT
	CAAGGCAGTCAAAGAAGGAACGTCGCTAAGAGACTTGGATATGTTCACC
	AAGCTTCTATCGGAGCTCTAA
CtOGT2	ATGGAAGTTCACATAGCCATTTTTCCGAGTCCAGGGATGGGTCACCTCAT
	TCCACTCACTGAGTTAACTCGCCAACTCCTCCGCCTCCTCCACTGTCGAG
	TCTTCATCACCTTCATCATTCCCACCACCGCCGGAGCTCCGATTAAACCC
	CAAAATGACATCCTCAACGCCATGCCGGAAAACGTAACTTCCGTTTTCCT
	TCCGCCCGTAGATCTAAACGACCTTCCACCGGATGCTTCCCTCGGGGCTC
	GGATATCAATCACTTTGACTCGGTCGTTACCCGCCCTACGTCAAACTCTC
	TCTGAGTTAACTCATGACTCGACTCGTAAACGTCCCTCGGCTTTGGTTGTT
	GACATTTTCGGCCCTCCTAGTTTCGAAATTGCTAAAGAATTCCATATTTCA

CCCTATATTTTCTCCACGGTTTCAGCTATGGTGCTGGTTAGCATCTTCCAT
ACACCTCTTCTACATGAGATGTTTGCTGGTGGTTTAGGTGAACCGGTCAC
ACTTCCAGGATGTGTTCCGGTTCAACCAACCGATTTCCCTGTACGGGCTC
ACCATTATGACCCAATTGTGCTGTGTAAGATGTATAATTCCGCAAAGGGT
ATACTTGTAAACAGCTTTGTTGAACTTGAACCGGGTGCCTTTAAAGCCAT
GGAAGAAGGTGAGTGGTGTAAACCTGATATTTTACCGGTCGGACCGTTG
ATACGCACCGGTTCAGAACAACAAGCCGACGACGGGTTTGAATGTCTGA
AGTGGTTGGATAAGCATCCCGTCGGGTCGGTTTTATTTGTCTCATTCGGAA
GTGGTGGCACATTGTCCCAGAAACAACTCGATGAACTTGCATTCGGGTTG
GAGAAAAGTGGTCAAAGATTTATTTGGGCCGTAAAAAGCCCAAGTGAAA
AAGTAAATGCATGTTACTTCAGTGCAAAAGGCGAACTCGACCCATTTATT
TTTTTACCCAATGGGTTCTTGGATCGGGTCAGGGACCGTGGTCTGGTCCTG
TCGTCGTGGGTCCCTCAGGTTGAGATCCTGGGTCACAGCTCAACCGGAGG
GTTTCTGACGCACTGCGGCTGGAACTCCATTCTCGAGAGCATTGCAAATA
GAGTTCCAATGATTGCTTGGCCGCTTTACGCGGAGCAACGAATGAATGCT
GTGAATTTGACAGATGGTTTAGGTGTCGGTTATCGGGTAAAAGTGGGTGA
AAATGGATTGGTGGGAAGAGATGAAATCGAGAAATGTGTTAGAAGTTTA
ATTGAGGGAGAAGATGGAGTTAAGATGAGGACGAAAATGGTTGAACTG
AAAGCGGGTGGTGCAATGGCGTTGAGCCAAGATGGTTCATCAACAAGGG
CAATCTTACACGTGGCTAAAAAGTGGGCTGAGTAA

Figure S1. (a) The three-dimensional protein structure prediction of *CtOGT1*; (b) The three-dimensional

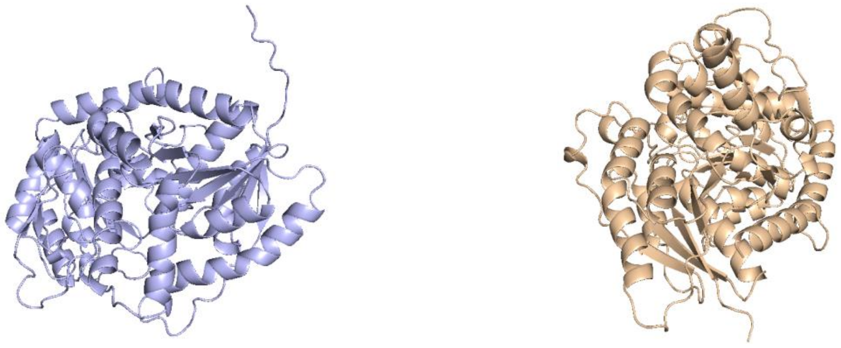

(**a**) (**b**)

protein structure prediction of *CtOGT2*.

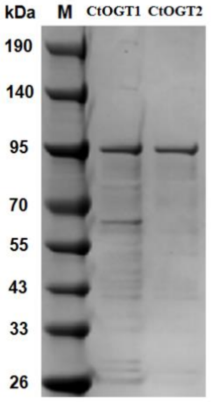

Figure S2. SDS-PAGE analysis of recombinant *CtOGT1* and *CtOGT2* purified by affinity chromatography. Note: M: Marker.

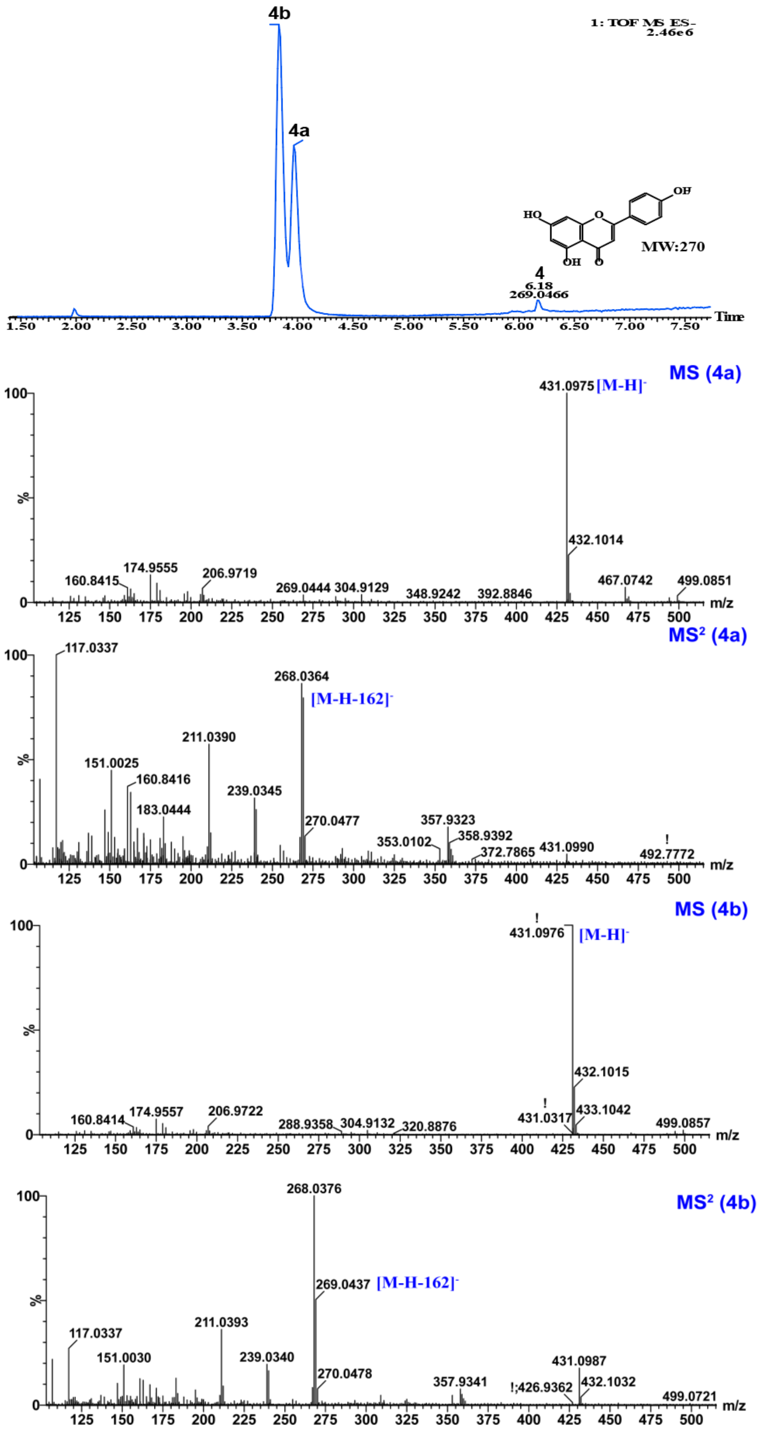

Figure S3. LC-MS analysis of *CtOGT1* and *CtOGT2* catalyzed product using apigenin as the substrate.

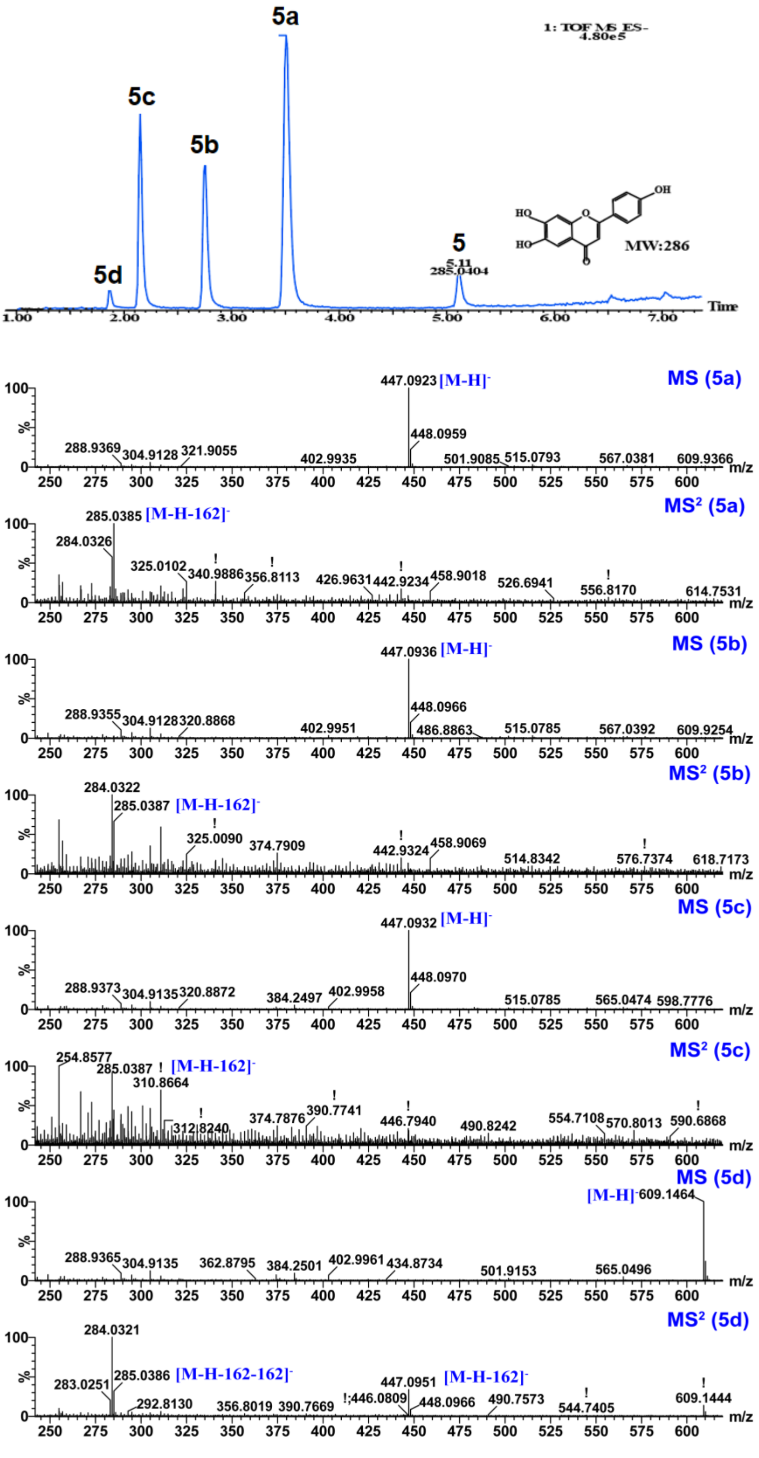

Figure S4. LC-MS analysis of *CtOGT1* and *CtOGT2* catalyzed product using scutellarein as the substrate.

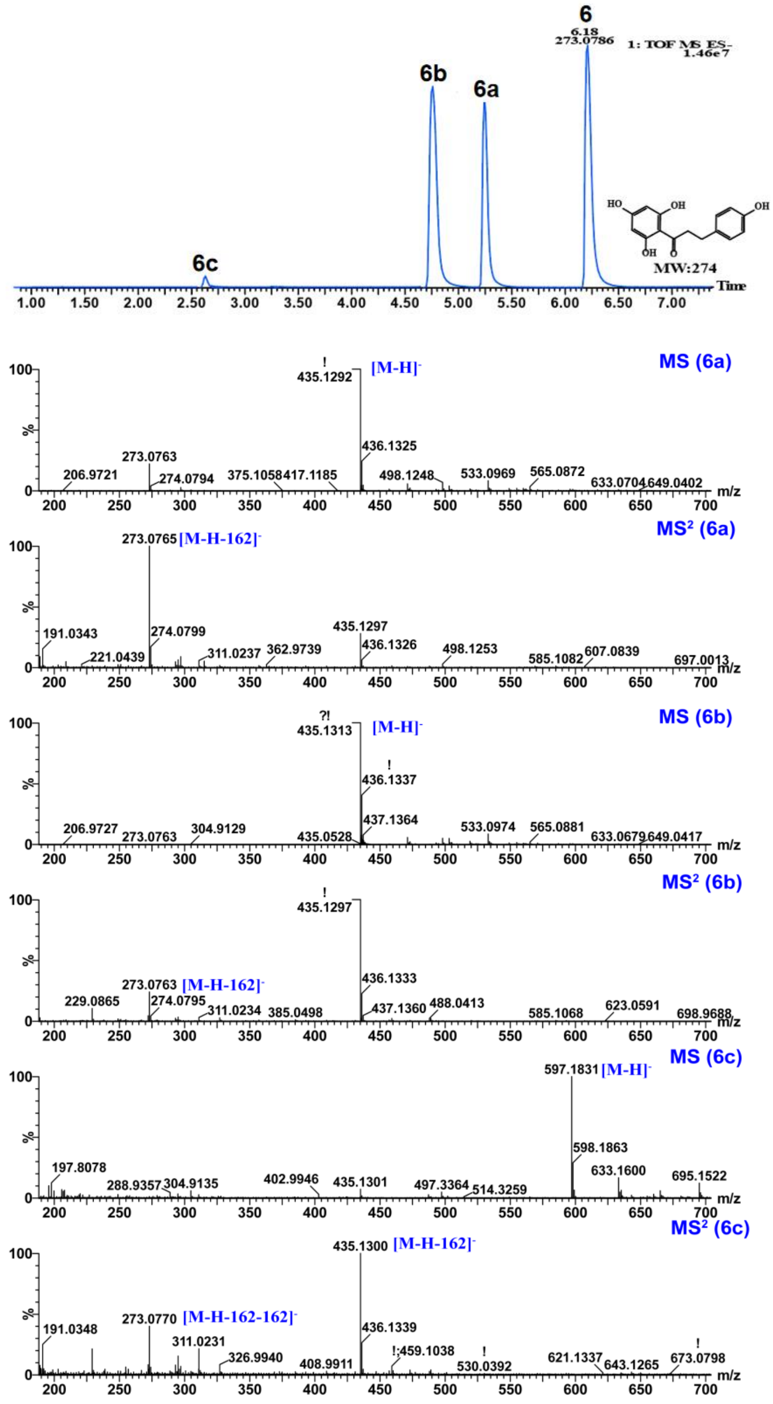

Figure S5. LC-MS analysis of *CtOGT1* and *CtOGT2* catalyzed product using phloretin as the substrate.

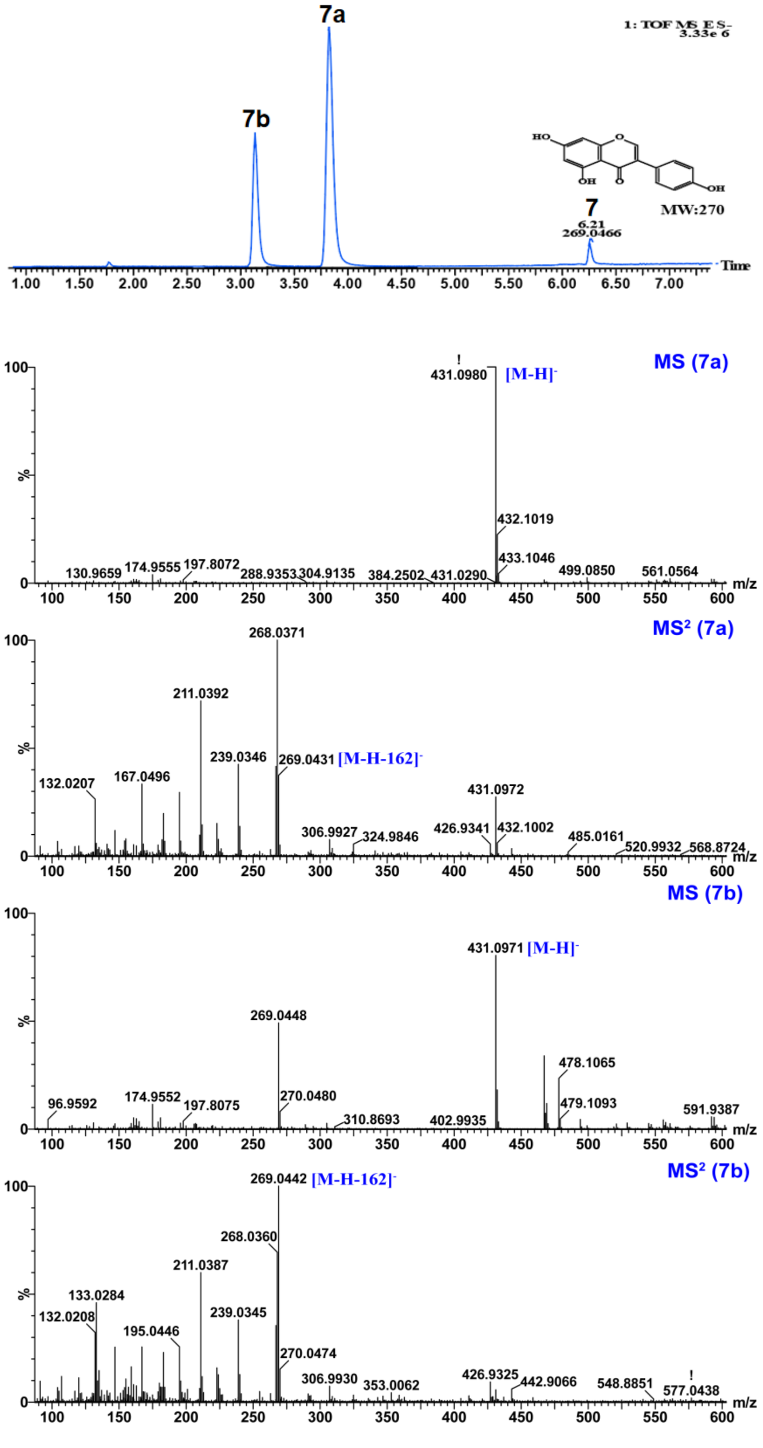

Figure S6. LC-MS analysis of *CtOGT1* and *CtOGT2* catalyzed product using genistein as the substrate.

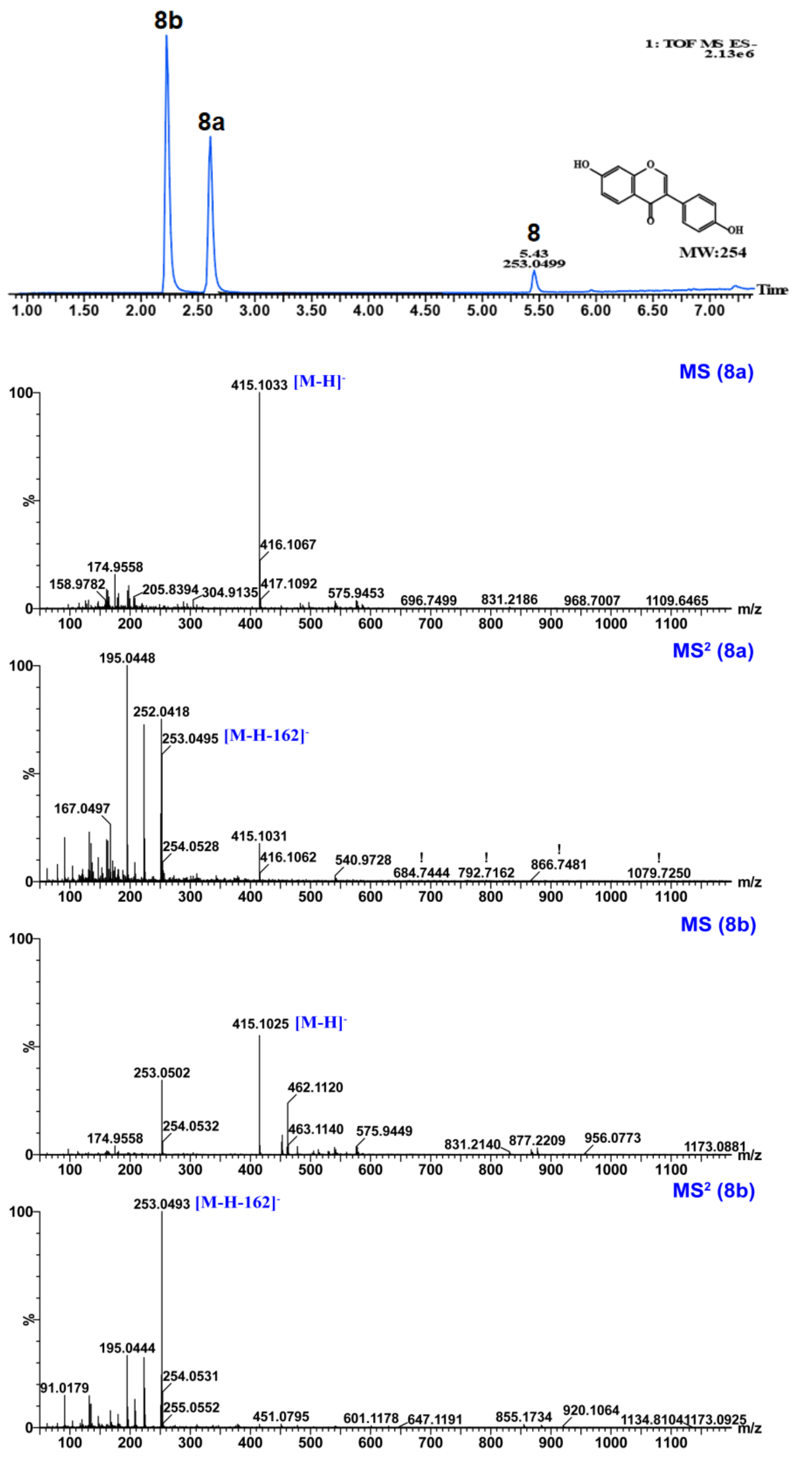

Figure S7. LC-MS analysis of *CtOGT1* and *CtOGT2* catalyzed product using daidzein as the substrate.

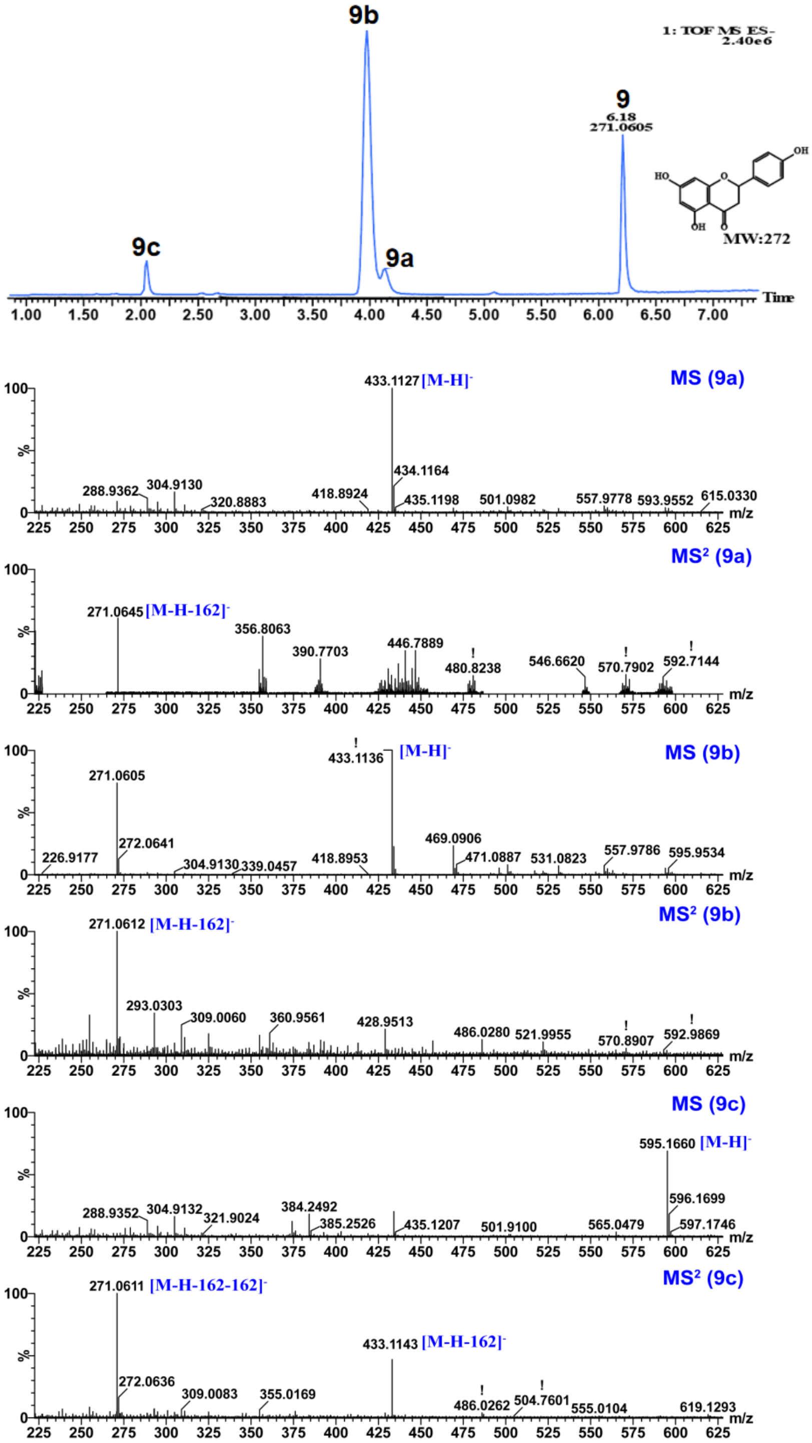

Figure S8. LC-MS analysis of *CtOGT1* and *CtOGT2* catalyzed product using naringenin as the substrate.

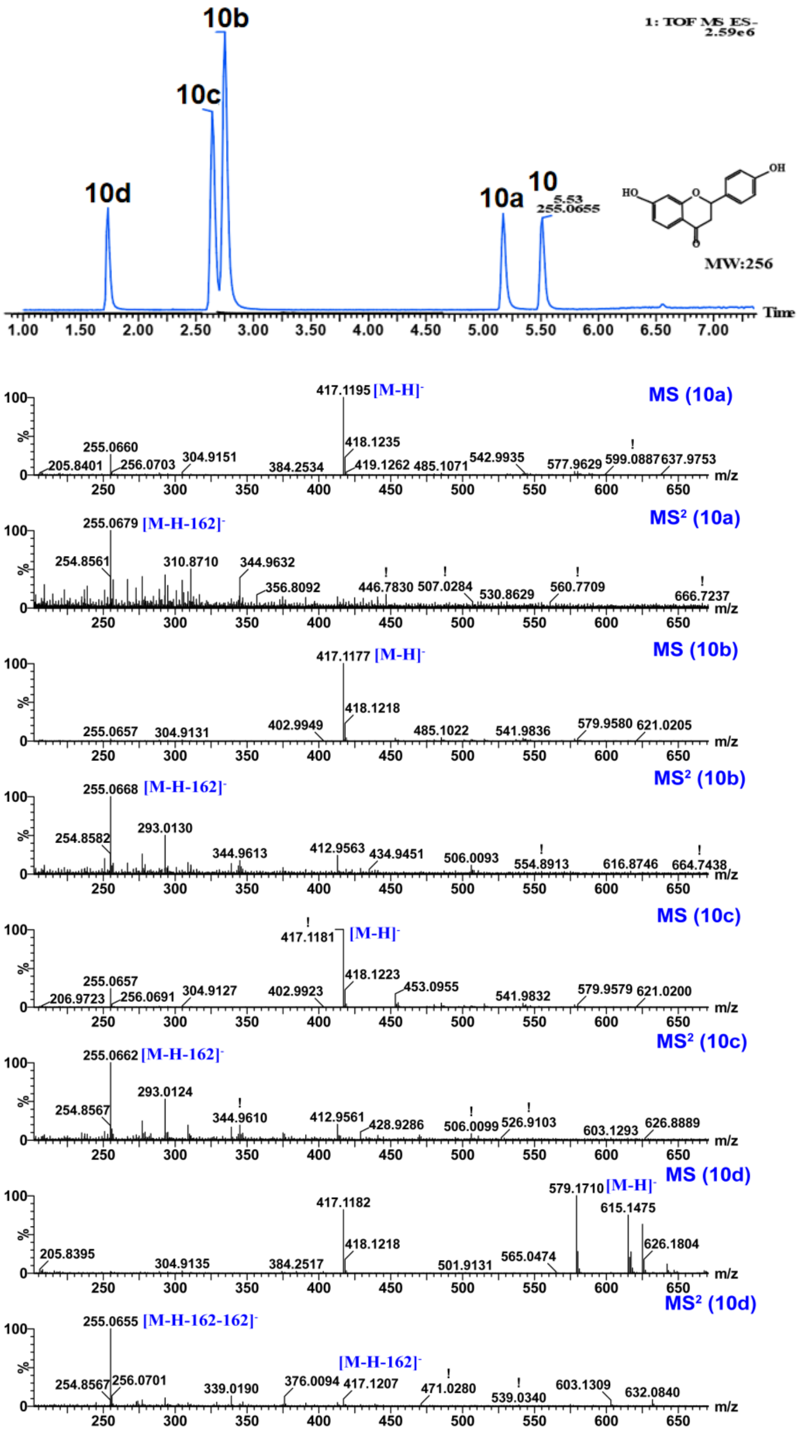

Figure S9. LC-MS analysis of *CtOGT1* and *CtOGT2* catalyzed product using glycyrrhizin as the substrate.

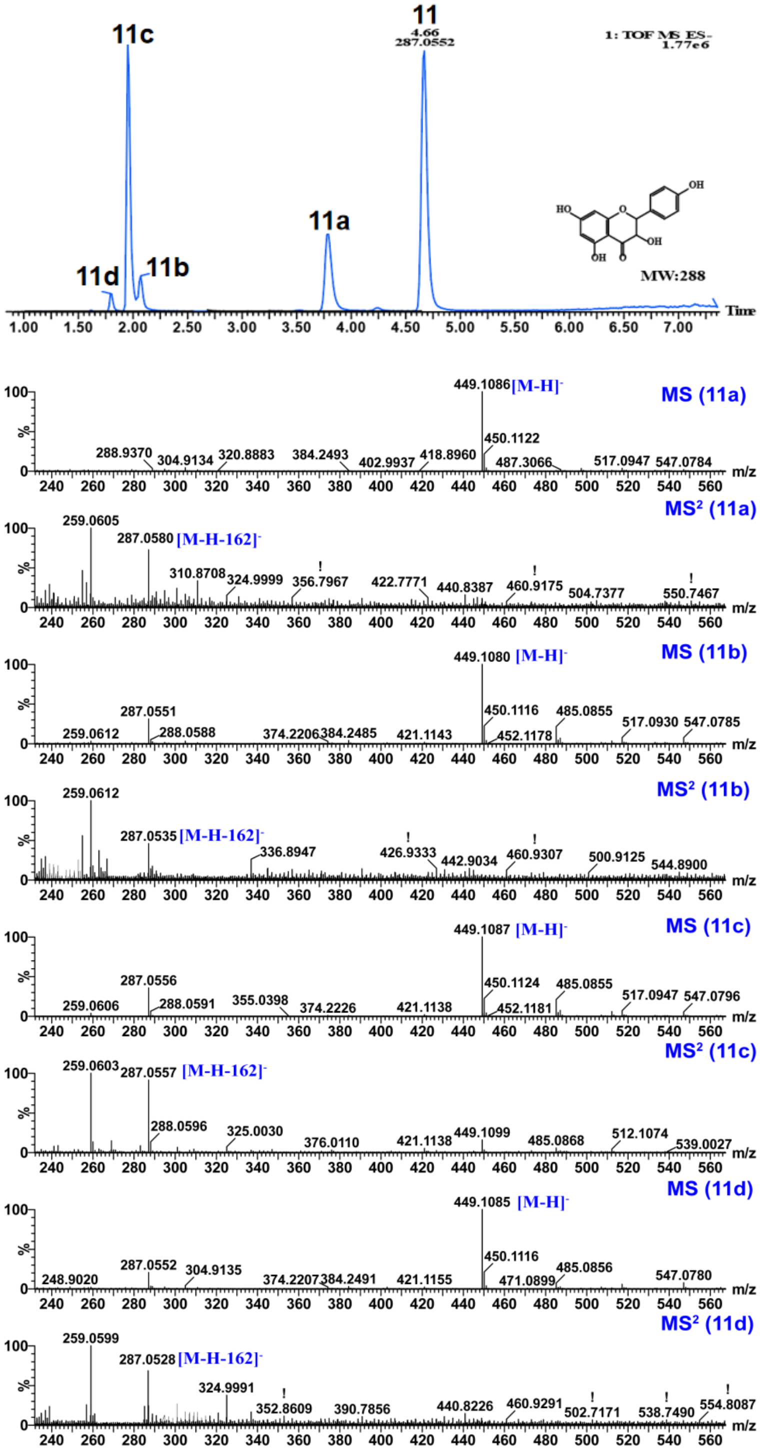

Figure S10. LC-MS analysis of *CtOGT1* and *CtOGT2* catalyzed product using dihydrokaempferol as the substrate.
